# Effect of loading rate and pH on glycerol fermentation and microbial population in an upflow anaerobic filter reactor

**DOI:** 10.1007/s00449-024-03003-6

**Published:** 2024-06-01

**Authors:** Cândida N. Cordeiro, Patricia Rojas, Shyrlane T. S. Veras, Mario T. Kato, Lourdinha Florencio, José Luis Sanz

**Affiliations:** 1 Department of Molecular Biology, Autonomous University of Madrid, 28049 Madrid, Spain; 2 Department of Civil and Environmental Engineering, Laboratory of Environmental Sanitation, Federal University of Pernambuco, Recife, PE 50740-530 Brazil

**Keywords:** 1,3-propanediol, Ethanol, Silicone support, Biofilm, *Clostridium*, *Lacrimispora*, Microbial consortia

## Abstract

A reactor with silicone tubes as support medium was used for glycerol fermentation. The experimental set-up consisted of three phases. In P1, the applied glycerol loading rate (gly-LR) was in the range of 6–10 g.L^−1^.d^−1^ at an influent pH of 7.9 ± 0.4. In P2, gly-LR was kept constant (18.0 ± 1.8 g.L^−1^.d^−1^) with different doses of NaHCO_3_. Finally in P3, two different gly-LR (9 and 18 g.L^−1^.d^−1^) were evaluated, dosing 1 g-NaHCO_3_ per g-COD of glycerol. Glycerol consumption was close 90%. The main end-product was 1,3-propanediol (1,3-PDO) (0.40 mol.mol-gly^−1^), but ethanol was also generated, particularly at pH above 8 and low gly-LR (0.20 mol.mol-gly^−1^). After 1-year operation with glycerol as the only carbon source, a drastic shift in the bacterial community was observed. The 1,3-PDO producers *Lacrimispora* and *Clostridium* became dominant, although non-glycerol-degrading fermentative genera, e.g., *Actinomyces* and *Eubacterium*, thrived at the expense of cellular breakdown products.

**Graphical abstract:**

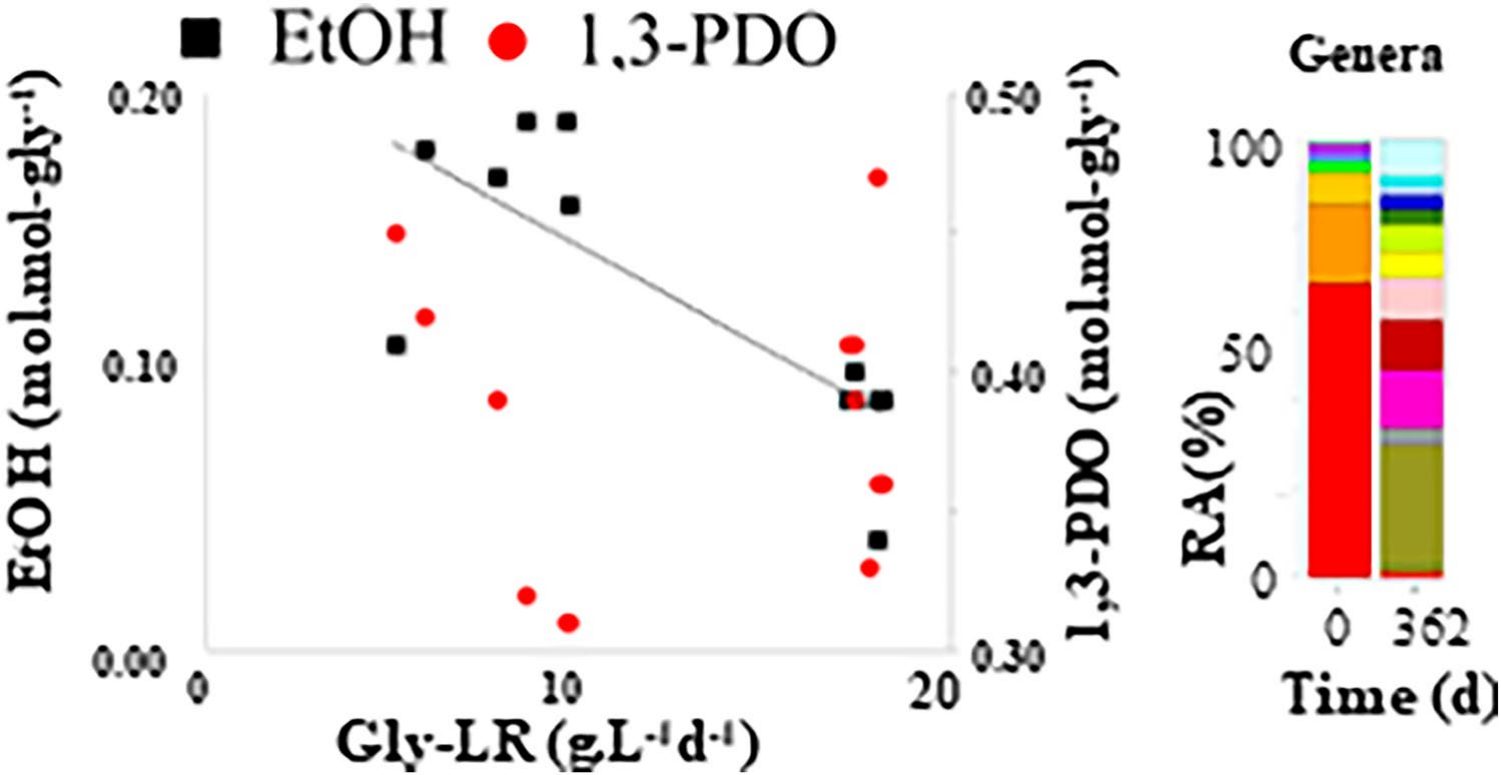

**Supplementary Information:**

The online version contains supplementary material available at 10.1007/s00449-024-03003-6.

## Introduction

Biodiesel production has increased as an alternative to dependence on petroleum derivatives. However, it generates large volumes of glycerol, around 10% (w/w) of the biodiesel produced, which has a low economic value and needs solutions for its recovery and valorization [1].

Glycerol can be biologically converted into value-added products by fermentation, through two anaerobic metabolic pathways: the reductive one, in which 1,3-propanediol (1,3-PDO) is the major product, and the oxidative one, in which H_2_, ethanol, butanol, and short-chain organic acids are produced in different ratios depending on the bacteria involved [2]. Glycerol has higher degree of reduction than other carbohydrates, such as glucose, so its fermentation can generate more reduced compounds, such as ethanol and 1,3-PDO, and fewer oxidized fatty acids, such as acetate. The yield of 1,3-PDO depends on the combination of both routes. When conditions are favourable for the reductive pathway, glycerol is first dehydrated to 3-hydroxypropanaldehyde, which is reduced to 1,3-PDO by consuming NADH_2_; and the yield is the highest when only acetic acid is obtained as a by-product [3–5]. On the other hand, the oxidative conversion of glycerol to pyruvate generates additional reducing equivalents that allow the co-production of ethanol and formic acid [6].

The metabolic pathway will depend on the micro-organism and the operating conditions applied. *Klebsiella*, *Clostridium*, and *Lactobacillus* are usually cited as 1,3-PDO-producers from glycerol [7, 8]. In addition, *Enterobacter* and *Klebsiella* are also involved in ethanol production [9–11], or even lactic acid [12] by glycerol fermentation.

Several studies have reported high yields of 1,3-PDO using pure cultures of microorganisms for glycerol fermentation. However, some requirements make scale-up difficult, such as the need for sterile conditions and complex nutrient media [5, 13, 14]. Glycerol fermentation using mixed microbial cultures is emerging as an alternative to eliminate these requirements, and also to achieve efficient co-production of 1,3-PDO and other valuable by-products [15, 16].

However, working with mixed cultures in continuous reactors involves challenges, such as competition between species and control of operating conditions to favour a particular metabolic pathway. The fermentation is influenced both by the dominant microorganisms and the operational conditions applied, such as pH, temperature, type of reactor, organic loading rate (OLR), and hydraulic retention time (HRT). Thus, the control of these parameters is of great importance in this biological process [17, 18]. Environmental stressors on 1,3-PDO biosynthesis and how they could be overcome using engineering strategies have been reviewed [19]. Immobilization of microorganisms in biofilms is considered as an operational strategy to improve the yield of desired products. Although further studies with immobilized biomass for the degradation of glycerol are still needed, high yields of 1,3-PDO have already been reported using the ceramic rings, pumice stones, polyurethane foams, and silicone hoses as support [8, 20–22].

Nevertheless, there are still not many studies reporting on the immobilization of mixed biomass and optimization of operating conditions for glycerol fermentation using reactors. Most of them have been performed in batch mode and using non-immobilized, pure, isolated, or genetically modified cultures, so the results obtained are not always applicable on a real scale [1, 17]. Therefore, to fill some of the above-mentioned gaps and to increase the knowledge on the effect of some operational parameters, especially glycerol loading rate (gly-LR), in the present work, an upflow anaerobic filter with a mixed culture immobilized on a silicone support was operated, evaluating the effect of applied gly-LR on the yields of 1,3-PDO and ethanol. The influence of the sodium bicarbonate dosage to provide enough alkalinity with the lowest operating costs was also studied.

## Materials and methods

### Medium composition

The mineral medium composition used was that in accordance with that described by Veras et al. [22], but initially without NaHCO_3_ addition. It contained macronutrients (g.L^−1^): K_2_HPO_4_.3H_2_O (3.4), KH_2_PO_4_ (1.3), NH_4_Cl (1.6), MgSO_4_.7H_2_O (0.2), CaCl_2_.2H_2_O (0.02), and FeSO_4_.7H_2_O (0.005) [23], and micronutrients according to [24]. Glycerol (97%, VWR Chemicals BDH Pro Lab^®^, Belgium) was used for inoculum activation, biofilm formation, and reactor feeding.

### Biofilm formation

The original inoculum was a mixed microbial consortium obtained from a glycerol-degrading reactor reported by [8]. Before its use in the present study, the biomass used as inoculum was maintained at 4 °C in a 1 L bottle containing crude glycerol and nutrients for about 2 years. The inoculum was then activated by consecutive transfers in 250 mL flasks, in which the 200 mL useful volume was filled with fresh medium (macro and micronutrients) and glycerol (10 g.L^−1^). In addition, 1 mL.L^−1^ of sodium sulphite solution (Na_2_S.9H_2_O, 100 g.L^−1^) was added to remove traces of dissolved oxygen. The flasks were sealed, and then purged with N_2_ and CO_2_ (80:20) for 3 min before the inoculation (1% v/v).

Two silicone hoses (Carl Roth^®^, Germany), with internal diameters of 0.4 and 0.3 cm, were used as support to promote the formation of biofilms. The feed solution containing nutrients and pure glycerol (initially at 30 g.L^−1^) was maintained at 4 °C and pumped through the hoses at a flow rate of 0.3 L.d^−1^ for 4 weeks. First, the feed solution was inoculated (2% v/v) with the suspension from the activation step, to promote biofilm formation. Then, during the last two weeks, the concentration of glycerol was changed to 15 g.L^−1^, and the hoses were fed without inoculum to confirm the glycerol consumption by the attached biofilm. The glycerol consumption and metabolite production were confirmed by analysis of daily effluent samples. The whole procedure was performed in a temperature-controlled room at 30 ± 2 °C.

### Reactor set-up and operation

After the biofilm formation, the hoses were cut into 2 cm pieces and taken to the 900 mL up-flow reactor, which was filled to about half its volume with them. Then, it was filled with nutrient medium until reaching the 800 mL useful volume. The HRT (days) was kept constant: 2.4 ± 0.7 (P1, days 0 to 197), 2.2 ± 0.3 (P2, days 198 to 334), and 2.1 ± 0.3 (P3, days 335 to 362). The reactor was fed initially with 15 g.L^−1^ of glycerol; the concentration was modified over the operational period depending on the desired gly-LR.

**Fig. 1:**
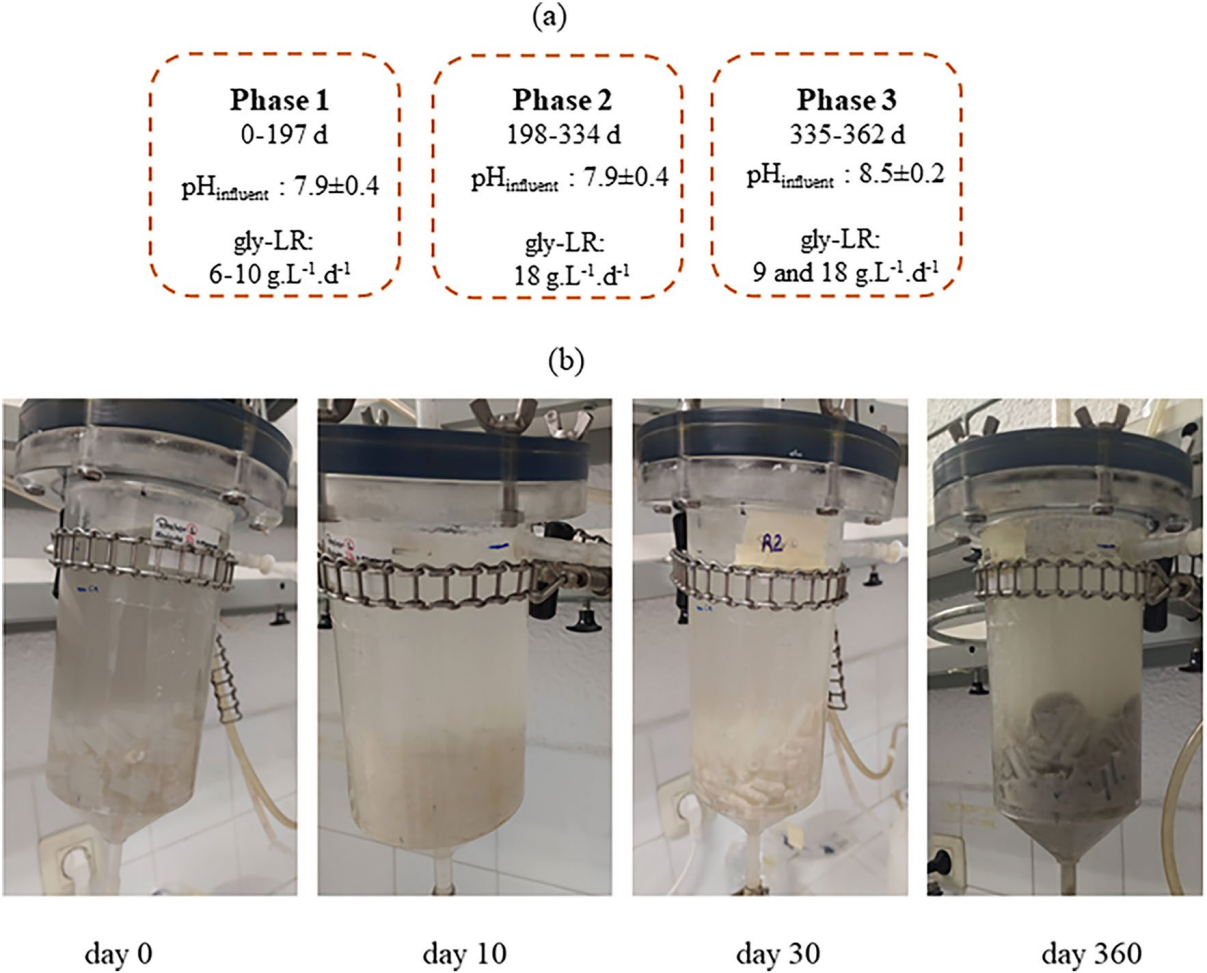
Diagram of the three phases into which the operation of the reactor can be divided (**a**). Biomass growth in the reactor over the 362 days of operation (images from the first 30 days and day 360 near the end) (**b**)

The reactor operational period (362 days) was divided into three phases (P): in P1, the applied gly-LR varied from 6 to 10 g.L^−1^.d ^−1^; in P2, gly-LR was maintained constant close to 18 g.L^−1^.d^−1^; in both phases, variable dosages of NaHCO_3_ as pH neutralizer were applied (Table 1); and in P3, two gly-LR (9 and 18 g.L^−1^.d^−1^) were applied, maintaining constant the amount of NaHCO_3_. The dosage of NaHCO_3_ was varied to (i) provide alkalinity and avoid a drop in reactor pH; (ii) to verify its influence on the yields of 1,3-PDO and ethanol. The setup is shown in Fig. 1a. The reactor was operated at a controlled temperature of 30 ± 2 °C. The daily monitoring consisted of measurements or control of the feeding flow, gly-LR, HRT, pH, and the fermentation products.

**Table 1:** Applied operational conditions and reactor performance in phases P1, P2, and P3

Phase	Time (d)	NaHCO_3_ (g.g COD^−1^)	pH_influent_	pH_effluent_	Gly-LR (g.L^−1^.d^−1^)	Gly consumption (%)	1,3-PDO (mol.mol^−1^)	EtOH (mol.mol^−1^)
P1	0–7	0	7.10 ± 0.30	4.47 ± 0.46	5.13 ± 1.53	39.60 ± 16.59	0.45 ± 0.09	0.11 ± 0.08
	8–24	1.00	8.04 ± 0.59	6.71 ± 1.49	5.88 ± 1.37	97.17 ± 1.61	0.42 ± 0.04	0.18 ± 0.07
	25–95	0.50	8.06 ± 0.22	7.14 ± 0.26	7.81 ± 1.31	94.97 ± 9.45	0.39 ± 0.06	0.17 ± 0.08
	96–133	0.50	8.05 ± 0.24	7.15 ± 0.25	9.68 ± 1.79	99.25 ± 2.57	0.31 ± 0.05	0.19 ± 0.04
	134–197	0.25	7.63 ± 0.28	6.41 ± 0.31	9.77 ± 1.90	99.13 ± 2.29	0.31 ± 0.08	0.16 ± 0.04
P2	198–252	0.50	8.10 ± 0.28	6.22 ± 0.50	18.22 ± 2.38	96.02 ± 8.34	0.36 ± 0.08	0.09 ± 0.03
	253–299	0.14	7.84 ± 0.22	5.54 ± 0.32	18.09 ± 1.24	91.00 ± 8.69	0.36 ± 0.04	0.09 ± 0.01
	300–305	0	7.02 ± 0.17	4.84 ± 0.43	17.84 ± 0.92	42.83 ± 3.32	0.33 ± 0.04	0
	306–313	0.14	7.88 ± 0.14	5.09 ± 0.13	17.28 ± 1.05	87.25 ± 4.03	0.41 ± 0.02	0.09 ± 0.01
	314–320	0.25	7.69 ± 0.17	5.28 ± 0.07	17.45 ± 0.34	91.93 ± 1.74	0.39 ± 0.04	0.10 ± 0.01
	321–334	0.50	8.22 ± 0.16	6.88 ± 0.72	17.43 ± 1.16	98.48 ± 1.80	0.41 ± 0.04	0.09 ± 0.01
P3	335–347	1.00	8.47 ± 0.21	7.66 ± 0.26	18.07 ± 1.49	91.84 ± 10.17	0.47 ± 0.07	0.04 ± 0.02
	348–362	1.00	8.34 ± 0.25	7.40 ± 0.19	8.61 ± 0.28	100	0.32 ± 0.10	0.19 ± 0.03

The concentrations of glycerol, alcohols, diols, and carboxylic acids in the liquid phase were quantified using a high-performance liquid chromatograph (HPLC 1200 Infinity Series, Agilent Technologies, USA) equipped with a refractive index detector (RID) and a MetaCarb 67H 300 × 6.5 mm column (Agilent Technologies, USA). The applied conditions were: temperature 40 °C (both column and detector), mobile phase 0.01 N H_2_SO_4_, flow rate 0.65 mL.min^−1^, and injection volume of 20 µL. The yields of metabolites were expressed in terms of moles of by-products generated per mole of glycerol consumed (mol.mol-gly^−1^).

### Microbial community analysis

Samples of biomass adhered to the support at the beginning (I) and end (F) of reactor operation were collected for extraction of bacterial metagenomic DNA using a FastDNA SPIN Kit for soil and FastPrep Instrument (MP Biomedicals, Santa Ana, USA). The concentration and quality of DNA were confirmed by measurement on an Invitrogen Qubit 4 Fluorometer (Thermo Fisher Scientific, USA).

Then, massive sequencing was performed using 341F/806R primers, and a MySEq V3 (2X300pb) platform (Illumina, San Diego, USA), by the FISABIO Sequencing and Bioinformatics Service (Valencia, Spain). The Mothur package v.1.47.0 (www.mothur.org) was used for sequence processing. The qualified sequences were clustered into operational taxonomic units (OTUs) defined by a 3% distance level based on the distance matrix and a bootstrap value higher than 60%. The SILVA 16S rRNA gene database was used for taxonomic classification. Confidence values < 80% were considered unclassified at the phylum level. Statistical analyses and graphical design were performed with the package vegan [25] for program R. The sequences obtained in this study has been registered in the National Center for Biotechnology Information (NCBI) under the BioProject accession number PRJNA934175 (https://www.ncbi.nlm.nih.gov/sra/PRJNA934175).

## Results

### Biofilm formation

During biofilm formation, glycerol consumption was between 50 and 70% and the effluent pH was about 5.0. The main product was 1,3-PDO with around 0.30 mol.mol-gly^−1^, followed by acetic acid and ethanol with approximately 0.10 mol.mol-gly^−1^ each. Glycerol consumption in the last two weeks of this period, even without inoculum added to the feed solution, confirmed that glycerol-consuming bacteria were already attached to the inner surface of the silicone tubes. During the reactor run time, biomass also grew on the outside surface of the silicone tubes (Fig. 1b).

### Influence of the operational conditions on the yields of 1,3-PDO and ethanol

**Fig. 2:**
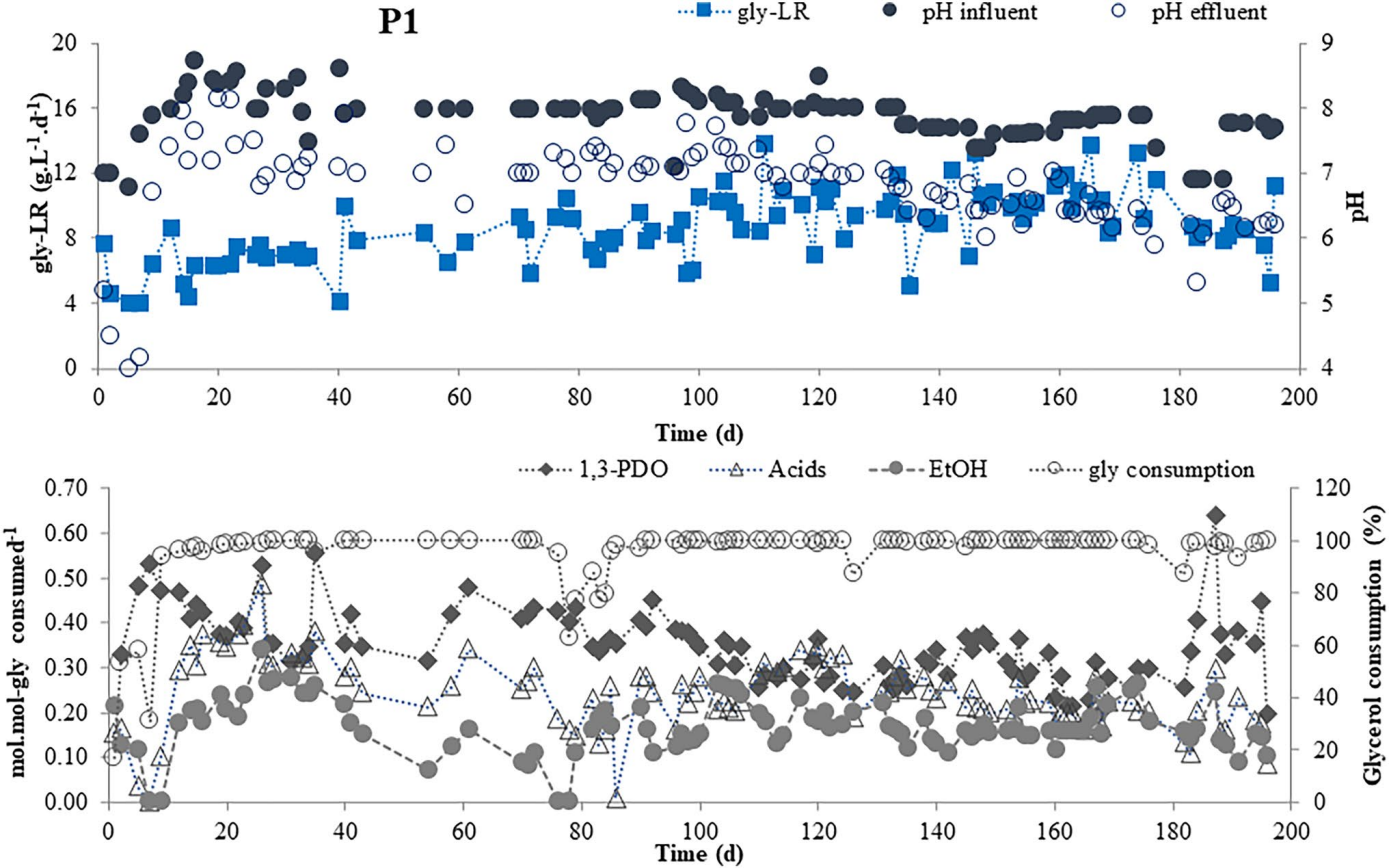
Applied glycerol loading rates (gly-LR), influent and effluent pH, glycerol consumption, and product yields (mol.mol-gly consumed.^−1^) during P1 phase (EtOH: ethanol)

In phase P1 (day 0 to 197), during the first 7 days of reactor operation, since no alkalinity was added, the pH dropped to 4.0, resulting in a decrease of glycerol consumption down to 31%. The dosage of 1 g NaHCO_3_.g COD^−1^ glycerol) after day 8 increased the ethanol and formate yields but decreased the 1,3-PDO and acetate yields. Therefore, the NaHCO_3_ dosage was diminished to 0.5 g.g COD^−1^ to assess its effect on the 1,3-PDO and ethanol yields. An increase of the gly-LR from about 6.0 to 8.0 g.L^−1^.d^−1^ (days 25 to 95) resulted in lower ethanol yields (day 30), accompanied by a slight increase of 1,3-PDO and acetate (Fig. 2). Then, after another small increase of the gly-LR to values close to 10 g.L^−1^.d^−1^, maintaining the same NaHCO_3_ dosage (0.5 g.g COD^−1^), the yields of 1,3-PDO and ethanol were not substantially affected. In addition, the decrease of bicarbonate by half (0.25 g.g COD^−1^, days 134–197) also had no substantial effect in the yields, thus making it possible to operate the reactor with less bicarbonate.

**Fig. 3:**
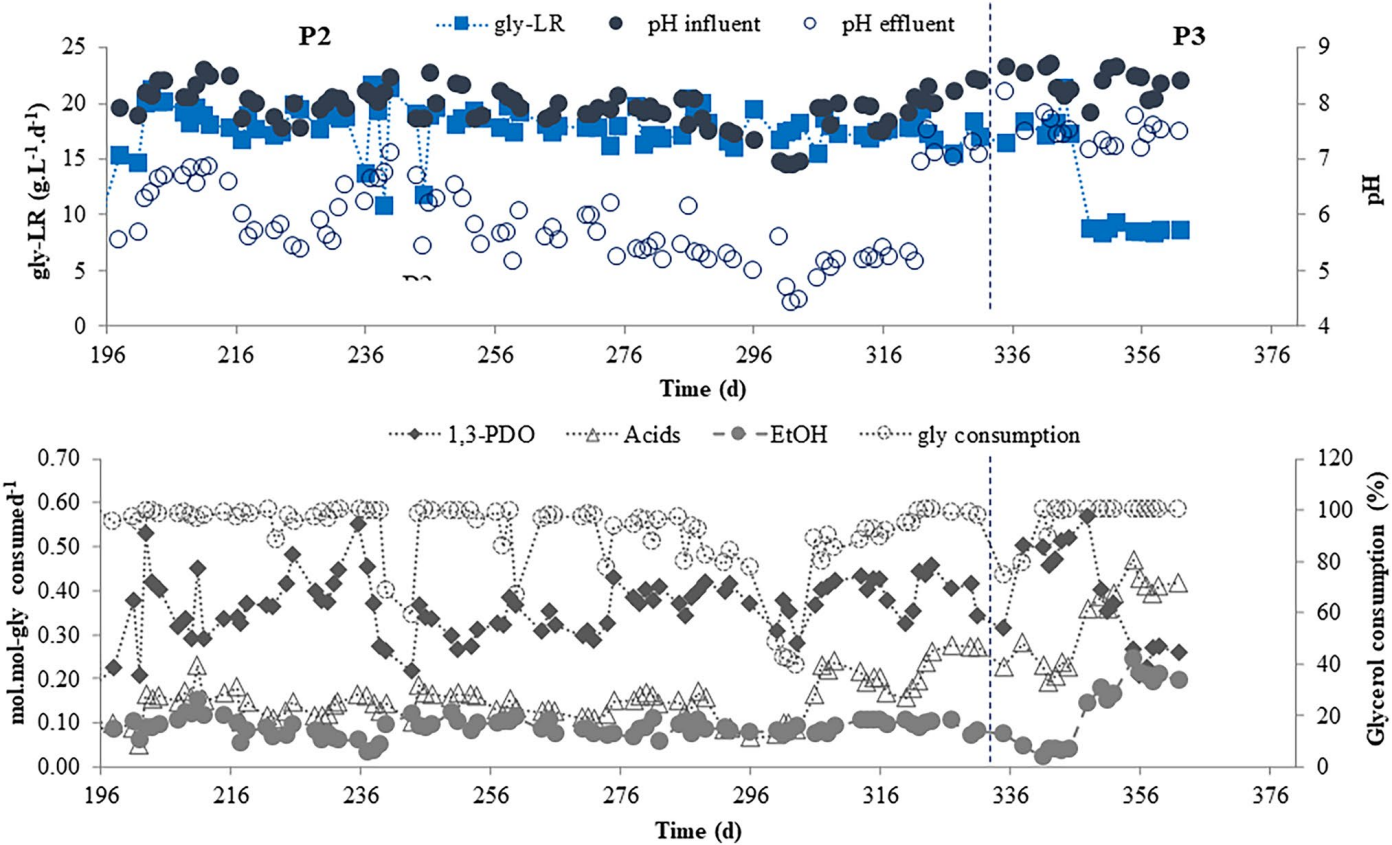
Applied glycerol loading rates (gly-LR), influent and effluent pH, glycerol consumption and product yields (mol.mol-gly consumed.^−1^) during P2 and P3 phases (EtOH: ethanol)

Therefore, the influence of decreasing NaHCO_3_ was studied in more detail during phase P2 (days 198 to 334), maintaining constant the gly-LR (18 g.L^−1^.d^−1^). The non-dosage of NaHCO_3_ (days 300–305) resulted in a sudden drop in the glycerol consumption, as already observed in the first 7 days of operation in P1, justifying the need to provide alkalinity to the reactor. The bicarbonate dosages tested in P2 varied from 0.5 to 0.14 g NaHCO_3_.g COD^−1^, which was the minimum considered enough to maintain the glycerol consumption above 87% (Fig. 3, Table 1).

**Fig. 4:**
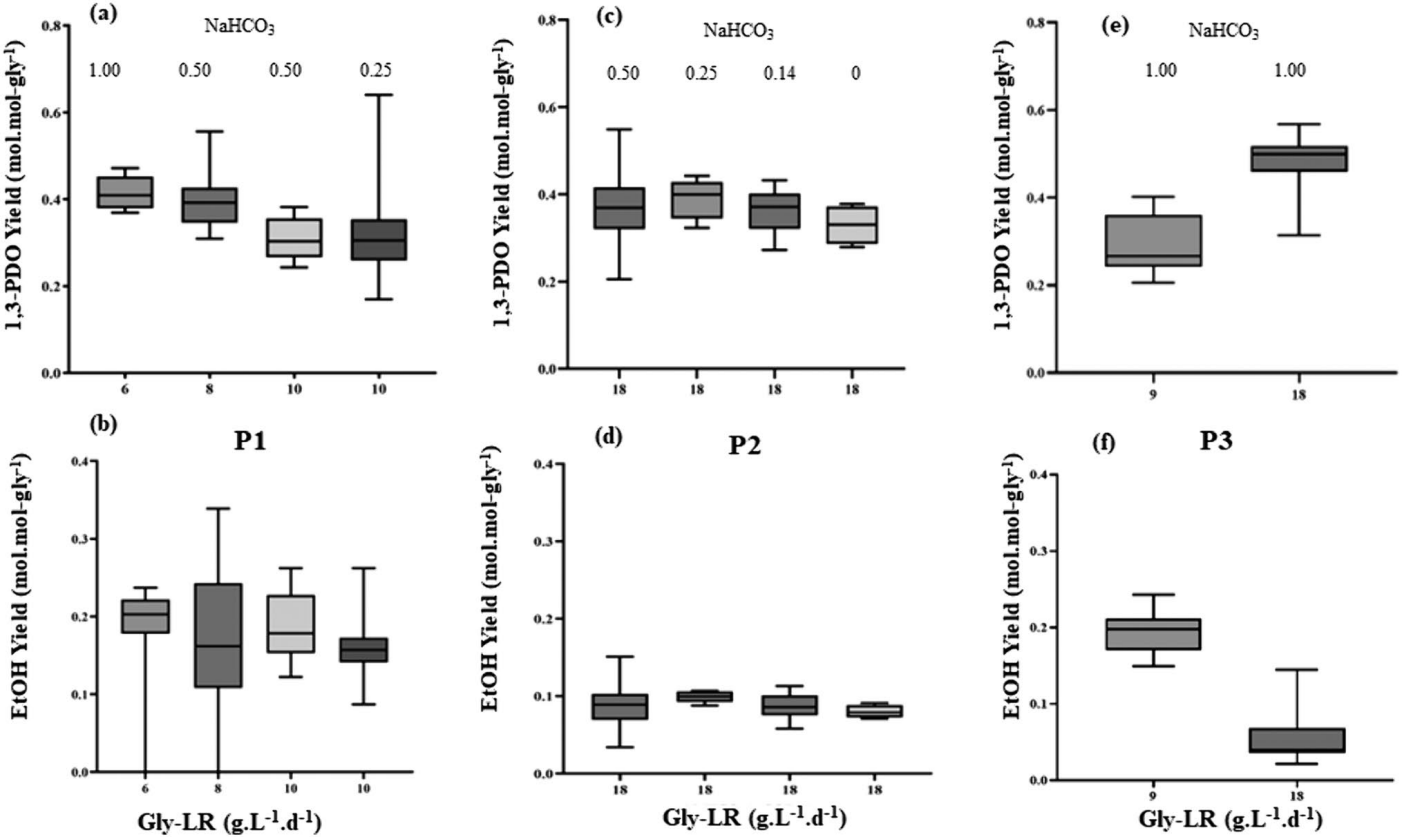
1,3-PDO yields during **a** P1, **c** P2, and **e** P3; ethanol (EtOH) yields during **b** P1, **d** P2, and **f** P3

Concerning the applied gly-LR, the results obtained in P1 and P2 led to the hypothesis that it would also be influencing the yields of ethanol and 1,3-PDO. Thus, in the last operational phase (P3), average rates of 18 and 9 g gly.L^−1^.d^−1^ were applied and provide enough alkalinity, 1.0 g NaHCO_3_ per g COD glycerol was added. Effectively, it was shown that increasing yields, from 0.04 to 0.19 mol of ethanol/mol glycerol (Fig. 4f, Table 1), were obtained when the gly-LR was diminished from 18 to 9 g gly.L^−1^d^−1^. Furthermore, the average yield of 1,3-PDO decreased from 0.47 to 0.32 mol.mol-gly^−1^ (Fig. 4e).

It is interesting that in phase P1, such bicarbonate influence could already be noticed. Comparing yields for the same applied gly-LR of about 10 g.L^−1^.d^−1^, but at two different dosages of 0.5 g.g COD^−1^ (days 96–133) and 0.25 g.g COD^−1^ (days 134–197), the resulting average yields of 1,3-PDO and ethanol did not differ (Fig. 4a, b). These results show that it would be interesting to enable a reactor operation with less added bicarbonate. In P2, NaHCO_3_ also did not influence the average yields of 1,3-PDO and ethanol (Fig. 4c, d).

**Fig. 5:**
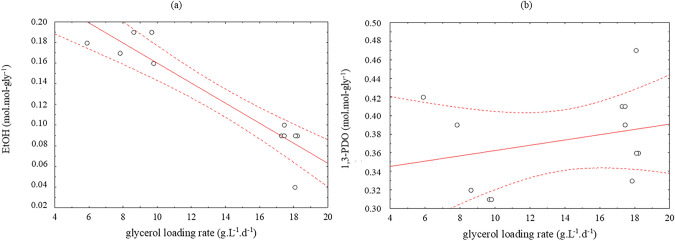
Correlation of glycerol loading rate with ethanol (EtOH) (**a**) and 1,3-PDO (**b**) molar yields. Solid line: fit line; Dashed lines: maximum error

Concerning the applied glycerol load, an inverse relationship between the gly-LR and the ethanol yields, was observed. In fact, the highest yields were reached at rates lower than 10 g.L^−1^.d^−1^ (Fig. 5a). Regarding 1,3-PDO, gly-LR had no significant influence on its yield (Fig. 5b).

According to the mass balance in terms of grams of COD per gram of glycerol consumed (Fig. SI1) and to the concentration of by-products in the effluent (Table SI1) 1,3-PDO was always the main product, but ethanol and volatile fatty acids (butyrate, acetate, formate) were significant, justifying the relatively low yield of 1,3-PDO, especially when gly-LR less than or equal to 10 g.L^−1^.d^−1^ was applied.

### Microbial community characterization

The Illumina system generated total reads (sequences) of 102,760 (I), and 81,646 (F). After removing the low-quality reads, 91,914 and 70,013 sequences, respectively, were considered for further analysis (Table 2). Their lengths, close to 450 bp, allow their reliable classification into OTUs with similarity higher than 97%, equivalent to species level. Only 6 and 11 genera of inoculum and reactor accounted for 98.6% and 89.5% of the total sequences, respectively. Moreover, the raretons (sequences present only one or twice) meant 99% of the total sequences were reliably assigned; and the Gini index was closed to 1 in both samples. These results pointed to the microbial community being dominated by a few genera carrying out most of the metabolic work. The Shannon index showed that the biodiversity increased from the inoculum (low) to the reactor (middle–high biodiversity) samples. This is confirmed by Shannon evenness and Simpson indexes (Table 2).

**Table 2:** Reads and richness, diversity, and evenness indices for the inoculum and reactor samples after 1-year operation

Index/sample	Inoculum	Reactor (final)
Total reads	102,760	81,646
Reliably assigned sequences	91,914	70,013
Average length (pb)	463	446
Number of OTUs/Sobs	991	1664
Raretons (%)	985 (99.4)	1583 (95.1)
Shannon	1.33 ± 0.01	2.88 ± 0.01
Good’s coverage (%)	99	98
Simpson	0.469 ± 0.003	0.122 ± 0.001
Shannon evenness	0.193	0.388
Gini	0.981	0.936

The index S_obs_, identical to the number of OTUs, the Good’s coverage index, close to 100%, and the rarefaction curves, which reached a plateau, pointed to a complete census of the bacteria present being achieved, in both the inoculum and reactor samples.

**Fig. 6:**
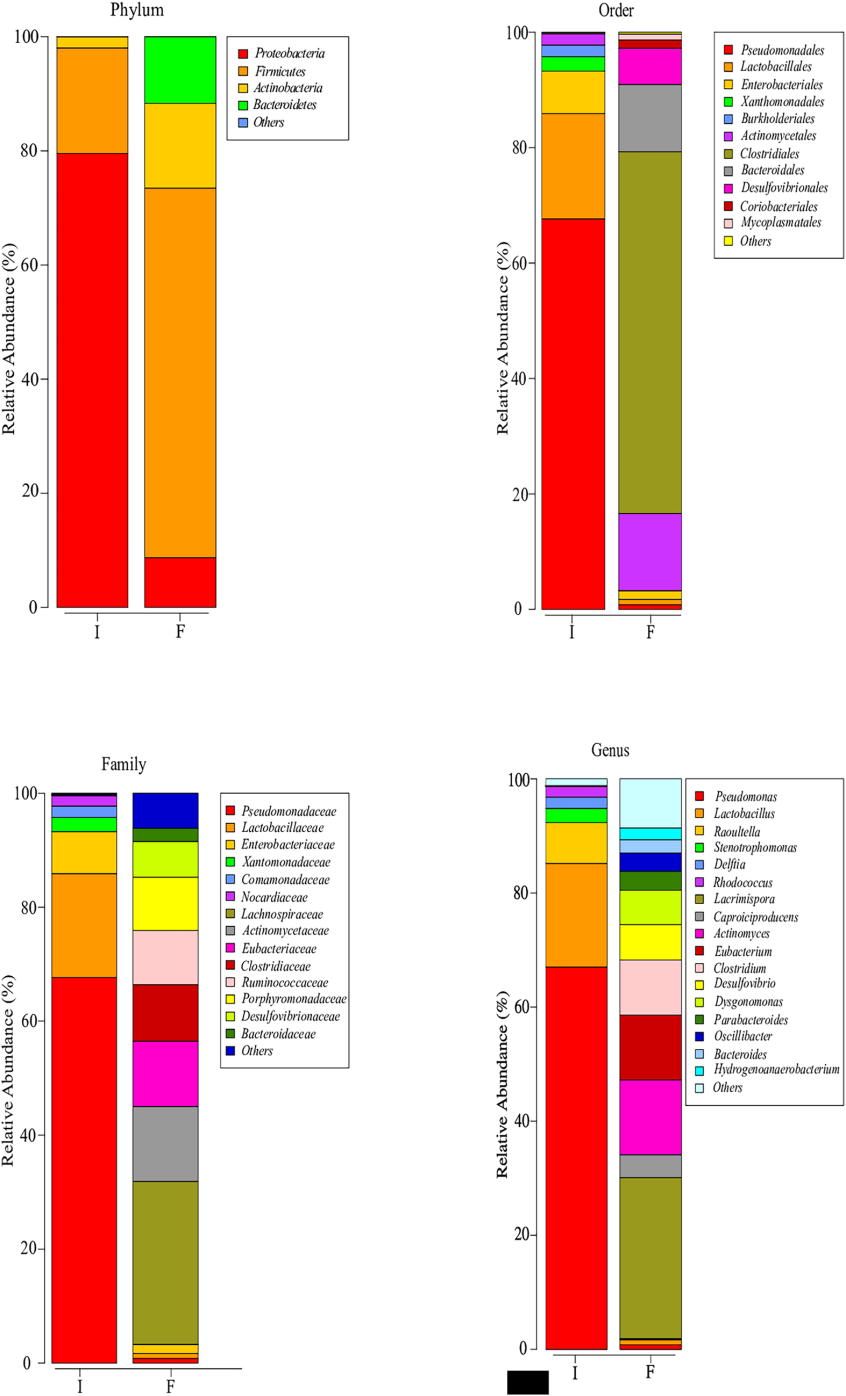
Taxonomic profiles at phylum (**a**), order (**b**), family (**c**), and genus (**d**). I: inoculum; F: final time of reactor operation. Taxa with coverage lower than 1% are grouped as Others

The biodiversity of the inoculum was very low: 98% of the total sequences reliably assigned were affiliated to only two phyla: *Proteobacteria* and, to a much lesser extent, *Firmicutes* (Fig. 6a). Within *Proteobacteria*, 67% of the total sequences retrieved belonged to the genus *Pseudomonas* (Fig. 6d and Table SI2), a chemoorganotroph aerobic microorganism.

After 1 year of operation, the bacterial population radically shifted. *Proteobacteria* drastically decreased in parallel with the increment of the phylum *Firmicutes*. While *Pseudomonas*, *Lactobacillus*, and *Raoultella* decreased to residual values (less than 1%), the order *Clostridiales* arose as the predominant microorganisms: from 0.2% to 62.6% of the total sequences (Fig. 6b, d, and Table SI2). The sequences affiliated to this order detected in the reactor were distributed among five different families, confirming the biodiversity of the bacteria present at the end of the operational period (Fig. 6c). At this time, the presence of the genera *Actinomyces* and *Dysgonomonas*, within the phylum *Actinobacteria*, and *Parabacteroides* and *Bacteroides*, within the phylum *Bacteroidetes*, was significant and, to a certain extent, unexpected (Fig. 6d, Table SI2).

## Discussion

In the present work, silicone tubes were applied as a support medium for the immobilization of the glycerol-degrading biomass. Silicone tubes provide physical (strength, porosity), chemical (hydrophilicity, reusability of the support), and mechanical stability properties which are important aspects of a support material for cell immobilization [21].

As for ethanol production, the highest yields were obtained when low gly-LR (8–9 g gly.L^−1^.d^−1^) and alkaline pH (above 8) were applied. The few studies on glycerol fermentation that report ethanol production almost always refer to pure cultures of glycerol-consuming bacteria. When a 2.5 oL fermenter was inoculated with isolated *Klebsiella* sp. HE1 to investigate the effect of pH in the range of 5.5–7.0 under glycerol concentrations of 10–70 g.L^−1^ [26], the authors reported the highest ethanol yields for the lowest glycerol concentrations and pH 6.0. Production of ethanol from crude glycerol at alkaline pH was studied for the first time by an isolated culture of *Klebsiella variicola* using test tubes containing 30 mL of glycerol and basal medium and varying the pH [9]. They reported that the best culture pH for ethanol production was 8.0–9.0, increasing glycerol consumption 1.4-fold at pH 9.0. When a CSTR was studied using mixed culture with dominance of *Klebsiella oxytoca*, under limiting concentrations of glycerol and pH 8, ethanol and formate yields were higher than 60% in terms of carbon removed [27]. Based on the results of the present work and those previously published, it can be concluded that if the priority objective is to obtain ethanol, the operation should be carried out with low glycerol loads and slightly alkaline pHs.

Most *Clostridium* species have the fastest growth at pH 6.5–7 [28]. Several studies have shown *Clostridium* spp. as glycerol fermenters and producers of 1,3-PDO [17, 29–33]. In the present study, the effluent pH was within the optimal range for *Clostridium* activity during most of the operational period, with 1,3-PDO being the main by-product. Concomitant with this work, additional experiments were conducted that resulted in ethanol yields close to 0.30 mol.mol-gly−1, when an alkaline effluent pH (8.5–8.8) and a low applied gly-LR (5.0–8.0 g.L^−1^.d^−1^) were maintained (data not shown). No 1,3-PDO production was observed except when pH was decreased to 8. Thus, it appears that this pH marks an upper limit for 1,3-PDO production. Additional experiments were carried out concomitantly with this work, resulting in ethanol yields close to 0.30 mol.mol-gly^−1^, when alkaline influent pH (8.5–8.8) and low applied gly-LR (5.0–8.0 g.L^−1^.d^−1^) were maintained. 1,3-PDO production was not observed (data not shown), except when the pH was diminished to 8. Thus, it appears that this fact marks a maximum pH limit for 1,3-PDO production.

Regarding the glycerol load applied, Fig. 5a shows its influence on the ethanol yields. The highest values were achieved when loads lower than 10 g.L^−1^.d^−1^ were applied. By varying the glycerol concentration from 4 to 25 g.L^−1^, it was observed that glycerol was a limiting factor, since ethanol yield decreased and 1,3-PDO yield increased when increasing glycerol concentration [27]. Similarly, the crude glycerol fermentation in batch tests by a pure culture of *Enterobacter* sp. showed that increasing the glycerol concentration from 5 to 10 g.L^−1^, the reductive pathway was favoured [34]. Obviously, if the aim is to produce 1,3-PDO, the applied conditions must be aimed at avoiding ethanol production.

The influence of glycerol loading in a UASB reactor with granular sludge on the H_2_, ethanol, and 1,3-PDO yields was evaluated [31]; when the loading rate increased from 25 to 50 g gly.L^−1^.d^−1^, the ethanol and 1,3-PDO production increased. Although their results are not comparable to those of this study, the authors demonstrated that the loading rate significantly influenced the metabolic pathways leading to glycerol degradation, resulting in different products generated. The authors related *Klebsiella pneumoniae* to the maximum production of 1,3-PDO and *Enterobacter* sp. with that of ethanol.

In summary, if the priority is to obtain ethanol, the operating conditions should be low gly-LR and slightly alkaline pH; but if the goal is to produce 1,3-PDO, those conditions must be avoided.

### Microbial community characterization

The inoculum presented a very low biodiversity and absolute predominance of the genus *Pseudomonas* (Fig. 6a, d, Table SI2). The presence of an aerobic chemoorganotroph microorganism was quite surprising, especially considering that the origin of the inoculum was an anaerobic glycerol-degrading reactor. Biomass from this reactor had been kept refrigerated for about 2 years. Although the best growth temperature for most *Pseudomonas* species is 28 °C, some species are psychrotolerant with a good growth rate at 4 °C [35]. Under these conditions, *Pseudomonas* could have thrived by utilizing the cellular decay products of other bacteria present in the biomass that were unable to survive for long under low temperature and oxygen conditions. The second predominant genus within *Proteobacteria* was *Raoultella* (7.1% of the total sequences), a facultative anaerobic enteric bacterium.

All the sequences retrieved from the inoculum, included in the phylum *Firmicutes*, were affiliated to the genus *Lactobacillus* (18.2%, Fig. 6d and Table SI2). The lactic acid bacteria are aerotolerant fermentative anaerobes. *Raoultella*, formerly *Klebsiella* [36], and *Lactobacillus* are typically glycerol-degrading bacteria, often detected in the conversion of glycerol to 1,3-PDO. Their presence in biomass extracted from a 1,3-PDO-producing reactor was expected and already reported [8, 13, 22, 37–41]. Their oxygen tolerance could justify their resilience in the inoculum. The two majority OTUs of the genus *Lactobacillus* have homology higher than 99% with *L. casei/paracasei* and *L. parabuchneri*, both heterofermentative, which could account for the ethanol detected in the phase P1 of the reactor.

The bacterial population changed dramatically after 1 year of reactor operation. While *Proteobacteria* decreased to less than 10%, the order *Clostridiales*, within the phylum *Firmicutes*, emerged as the predominant one (Fig. 6b, d, and Table SI2). In addition, the biodiversity increased markedly, as indicated by the diversity and evenness indexes. All genera belonging to the order *Clostridiales* are obligate anaerobes, usually chemoorganotrophs, gaining energy by fermentation, and producing a mixture of organic acids, such as lactate, formate, acetate, or butyrate. The sequences affiliated to this order detected in the reactor were distributed among five different families, showing the adaptation to the operating conditions of the reactor and the equitable role of several taxonomic groups (Shannon indices). The use of glycerol as the sole source of carbon and energy promoted the development of genera adapted to its degradation. *Lacrimispora* (28.2% of the total sequences retrieved from the reactor) showed 98.9% similarity to *Lacrimispora sphenoides*. *L. sphenoides* is a reclassification of *Clostridium sphenoides* [42] that was already reported in a work using a UASB reactor adapted to glycerol consumption [8]. Members of the genus *Clostridium* are commonly detected in glycerol-degrading reactors [17, 22, 30, 31]. *Clostridium pasteurianum* was the highly predominant bacterium (75.9%) found in the biomass attached to the silicone support in a 1,3-PDO-producing reactor fed with glycerol [8]. In the present study, 70% of the sequences affiliated to the genus *Clostridium* displayed 99.3% similarity to *C. pasteurianum*. Another prominent genus presents in the reactor, *Caproiciproducens*, is also considered capable or growing using glycerol [43].

Most of the sequences affiliated to the phyla *Actinobacteria* and *Bacteroidetes* were classified in the genera *Actinomyces*, *Dysgonomonas*, *Parabacteroides*, and *Bacteroides* (Fig. 6d, Table SI2). All they are chemoorganotrophs, with a fermentative metabolism. *Parabacteroides* and *Bacteroides* are obligate anaerobes, whereas *Actinobacteria* and *Dysgonomonas* are facultative anaerobes; except for *A. graevenitzii* and *A. neuii*, the use of glycerol has not been reported in these genera. Given that *Actinomyces* growth only occurs well in complex media containing rich biological ingredients [44], and that *Bacteroides* and its relatives are typical inhabitants of gut microbiota and wastewater, it can be considered that these bacteria could thrive in the reactor using compounds released by the cellular decomposition of bacteria that were unable to use glycerol as the sole source of carbon and energy.

In summary, the microbial community present in the inoculum underwent a drastic change because of the use of glycerol as the sole source of carbon and energy, and due to the operational conditions imposed on the reactor for 1 year. Dominant aerobic degrading organisms in the inoculum, e.g., *Pseudomonas*, disappeared and were replaced by anaerobic bacteria. Among these, there were genera well known to convert glycerol to 1,3-PDO, e.g. *Lacrimispora* and *Clostridium*, which accounted for 40% of the retrieved sequences. However, a significant part of the population consisted of non-glycerol-utilizing fermenting bacteria, well adapted to growth at the expense of products released by cellular decay of other bacteria. This could justify the relatively low yields of 1,3-PDO obtained, and the fermentation products, e.g., ethanol, formate, acetate, butyrate, and lactate, found in the reactor (Fig. SI1, Table SI1).

Due to the significant increase in global biodiesel production, an immense amount of glycerol needs to be removed. Furthermore, traditional chemical methods for obtaining 1,3-PDO are based on the catalytic conversion of ethylene oxide (Shell) or acrolein (Degussa) to 1,3-PDO. But chemical synthesis requires high temperature and pressure conditions, the use of chemical catalysts increasing the costs of the process, in addition to producing toxic intermediate compounds. The conversion of glycerol into 1,3-PDO by fermentation can be an economical alternative: the cost of the raw material is zero and the reactor requires a low energy input. However, the recovery of 1,3-PDO must be carried out with solvents, e.g. hexane, and the down-stream processes can be costly. Consequently, the improvement of these processes and yields of the desired by-products is mandatory. The present results are interesting for scale-up, as an immobilized complex microbial consortium showed robustness and adaptation to the varying operational conditions. Although the goal was to obtain 1,3-PDO as the main end-product, the trends observed for ethanol could be useful for its production if alkalinity conditions (pH above 8) and low loading rate were applied for this purpose. Since few species can ferment glycerol, and others could thrive using the compounds released by cell decay of bacteria unable to do so, further studies should be conducted to increase the yield clarifying the relationship of the dominant species with different pH, and the gly-LR, with the obtained products.

## Supplementary Information

Below is the link to the electronic supplementary material.

Supplementary file1 (DOC 92 KB)

Supplementary file2 (DOC 31 KB)

Supplementary file3 (DOC 48 KB)

## Data Availability

All data generated or analysed during this study are included in this published article.
