# Supplementary material for: Effect of loading rate and pH on glycerol fermentation and microbial population in an upflow anaerobic filter reactor
Source: Bioprocess Biosyst Eng. 2024 Jun 1;47(7):991–1002. doi: 10.1007/s00449-024-03003-6 (PMC11213801; doi:10.1007/s00449-024-03003-6)
Supplement: Supplementary file 1 — Supplementary file1 (DOC 92 KB) [file 449_2024_3003_MOESM1_ESM.doc]

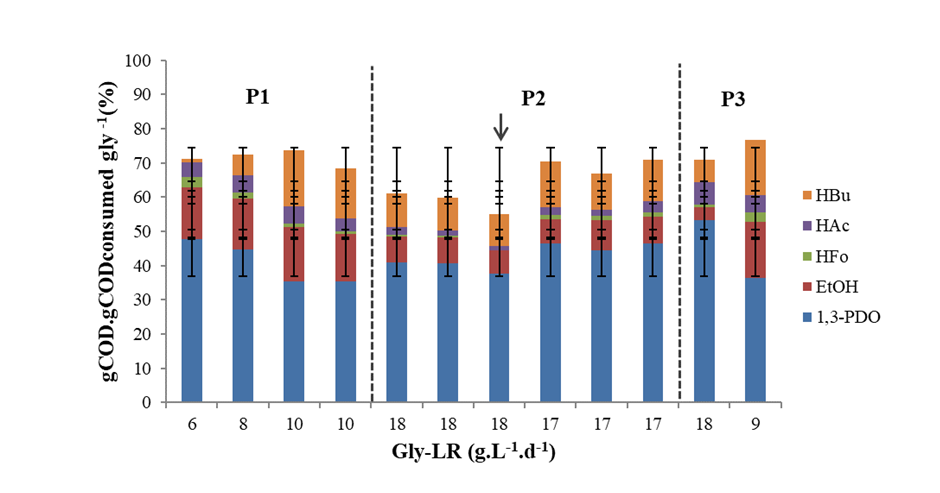


**Fig SI1**. Mass balance during the three operational phases (the arrow indicates the period in which NaHCO3 was not added to the medium; days 0 to 7 were not considered for the balance, as it was a period of adaptation of the reactor and of low efficiency (HBu: butyric acid; Hac: acetic acid; HFo: formic acid; EtHO: ethanol; 1,3-PDO: 1,3-propanediol). The COD of glycerol and by-products was determined using their concentrations quantified by HPLC and their theoretical COD.
