# Supplementary material for: Effect of loading rate and pH on glycerol fermentation and microbial population in an upflow anaerobic filter reactor
Source: Bioprocess Biosyst Eng. 2024 Jun 1;47(7):991–1002. doi: 10.1007/s00449-024-03003-6 (PMC11213801; doi:10.1007/s00449-024-03003-6)
Supplement: Supplementary file 2 — Supplementary file2 (DOC 31 KB) [file 449_2024_3003_MOESM2_ESM.doc]

**Table SI1. Concentration of glycerol fermentation products in the reactor**

| **Products in effluent (g.L^-1^)** | **Phase P1** | **Phase P2** | **Phase P3** |
| --- | --- | --- | --- |
| *Formate* | 0.68±0.52 | 0.67±0.37 | 1.21±0.37 |
| *Acetate* | 0.77±0.18 | 0.62±0.32 | 1.34±0.62 |
| *Butyrate* | 1.27±0.16 | 1.91±0.56 | 1.42±0.37 |
| *Ethanol* | 1.28±0.44 | 1.22±0.33 | 1.06±0.46 |
| *1,3-PDO* | 4.16±0.96 | 8.41±2.09 | 6.71±3.79 |
