# Supplementary material for: Effect of loading rate and pH on glycerol fermentation and microbial population in an upflow anaerobic filter reactor
Source: Bioprocess Biosyst Eng. 2024 Jun 1;47(7):991–1002. doi: 10.1007/s00449-024-03003-6 (PMC11213801; doi:10.1007/s00449-024-03003-6)
Supplement: Supplementary file 3 — Supplementary file3 (DOC 48 KB) [file 449_2024_3003_MOESM3_ESM.doc]

**Table SI2** Phylogenetic relationships of the taxa found in the inoculum and at the end of the reactor operation. The % values are the percentage of the sequences retrieved from the inoculum (I) and from the reactor (F) with respect to the total number of reliably assigned sequences

| **PHYLUM** | % | **ORDER** | % | **FAMILY** | % | **GENUS** | % |
| --- | --- | --- | --- | --- | --- | --- | --- |
| ***Proteobacteria*** | I: 79.6  F: 8.7 | *Pseudomonadales* | I: 67.6  F: 0.8 | *Pseudomonadaceae* | I: 67.6  F: 0.8 | *Pseudomonas* | I: 67.0  F: 0.8 |
|  |  | *Enterobacteriales* | I: 7.4  F: 1.5 | *Enterobacteriaceae* | I: 7.4  F: 1.5 | *Raoultella* | I: 7.1  F: 0.1 |
|  |  |  |  |  |  | *Klebsiella* | I: 0  F: 1.3 |
|  |  | *Xanthomonadales* | I: 2.5  F: 0 | *Xhantomonadaceae* | I: 2.5  F: 0 | *Stenotrophomonas* | I: 2.5  F: 0 |
|  |  | *Burkholderiales* | I: 2.0  F: 0 | *Comamonadaceae* | I: 2.0  F: 0 | *Delftia* | I: 2  F: 0 |
|  |  | *Desulfovibrionales* | I: 0  F: 6.3 | *Desulfovibrionaceae* | I: 0  F: 6.3 | *Desulfovibrio* | I: 0  F: 6.2 |
| ***Firmicutes*** | I: 18.5  F: 64.8 | *Lactobacillales* | I: 18.2  F: 0.9 | *Lactobacillaceae* | I: 18.2  F: 0.9 | *Lactobacillus* | I: 18.2  F: 0.9 |
|  |  | *Clostridiales* | I: 0.2  F: 62.6 | *Lachnospiraceae* | I: 0  F: 28.2 | *Lacrimispora* | I: 0  F: 28.2 |
|  |  |  |  | *Eubacteriaceae* | I: 0.1  F: 11.5 | *Eubacterium* | I: 0.1  F: 11.4 |
|  |  |  |  | *Clostridiaceae* | I: 0.1  F: 9.9 | *Clostridium* | I: 0.1  F: 9.6 |
|  |  |  |  | *Ruminococcaceae* | I: 0  F: 9.5 | *Caproiciproducens* | I: 0  F: 4.2 |
|  |  |  |  |  |  | *Oscillibacter* | I: 0  F: 3.2 |
|  |  |  |  |  |  | *Hydrogenoanaero-bacterium* | I: 0  F: 2.1 |
|  |  |  |  | Fam_XI_Incertae_se | I: 0  F: 1.5 | *Tissierella* | I: 0  F: 1.5 |
| ***Actinobacteria*** | I: 2.0  F: 14.8 | *Actinomycetales* | I: 2.0  F: 13.4 | *Nocardiaceae* | I: 1.8  F: 0.1 | *Rhodococcus* | I: 1.8  F: 0.1 |
|  |  |  |  | *Actinomycetaceae* | I: 0  F:13.1 | *Actinomyces* | I: 0  F: 13.1 |
|  |  | *Coriobacteriales* | I: 0  F: 1.5 | *Coriobacteriaceae* | I: 0  F: 1.5 | *Adlercreutzia* | I: 0  F: 1.4 |
| ***Bacteroidetes*** | I: 0  F: 11.7 | *Bacteroidales* | I: 0  F: 11.7 | *Porphyromonadaceae* | I: 0  F: 9.3 | *Dysgonomonas* | I: 0  F: 6.3 |
|  |  |  |  |  |  | *Parabacteroides* | I: 0  F: 3.3 |
|  |  |  |  | *Bacteroidaceae* | I: 0  F: 2.3 | *Bacteroides* | I: 0  F: 2.3 |
